# Validation of Residual Cancer Burden as Prognostic Factor for Breast Cancer Patients After Neoadjuvant Therapy

**DOI:** 10.1245/s10434-019-07741-w

**Published:** 2019-08-26

**Authors:** Hannah Deborah Müller, Florian Posch, Christoph Suppan, Ute Bargfrieder, Melanie Gumpoldsberger, Robert Hammer, Hubert Hauser, Nadia Dandachi, Kurt Prein, Herbert Stoeger, Sigurd Lax, Marija Balic

**Affiliations:** 1 Division of Oncology, Department of Internal Medicine, Medical University of Graz, Graz, Austria; 2 Comprehensive Cancer Center Graz, Medical University of Graz, Graz, Austria; 3 Department of Pathology, Hospital Graz South-West, Graz, Austria; 4 Department of Surgery, Hospital Graz South-West, Graz, Austria; 5 Institute of Pathology, Medical University of Graz, Graz, Austria; 6 Department of Pathology, Johannes Keppler Univeristy Linz, Linz, Austria

## Abstract

**Background:**

Assessing the residual cancer burden (RCB) predictive performance, the potential subgroup effects, and time-dependent impact on breast cancer patients who underwent neoadjuvant therapy in a developer’s independent cohort is essential for its usage in clinical routine.

**Methods:**

Between 2011 and 2016, the RCB scores of 184 female breast cancer patients were prospectively collected, and subsequent clinicopathological and follow-up data were obtained retrospectively. Recurrence-free survival (RFS), overall survival (OS), as well as subgroup analysis, and time-dependent variables were calculated with multivariate, complex, or linear statistical models.

**Results:**

A total of 184 patients (HER2 33%, TNBC 27%), with a mean follow-up time of 4 years, treated with neoadjuvant systemic therapy (92% anthracycline-taxane based) were analyzed revealing 43 events (38 recurrences, 28 deaths). High RCB scores were associated with recurrence (median index: 2.34 vs. 1.39 points, rank-sum *p* < 0.0001), decreased RFS (hazard ratio [HR] = 1.80, 95% confidence interval [CI] 1.44–2.24, *p* < 0.0001) and reduced OS (HR 1.96, 95% CI 1.49–2.59, *p* < 0.0001). The RCB score showed proportionality of hazards (interaction HR with linear follow-up time = 1.00, *p* = 0.896) and good discriminating power (Harrell’s c index 0.7).

**Conclusions:**

Our results confirm the RCB score as externally valid prognostic marker and being independent of molecular subtype for RFS and OS in a clinical setting.

**Electronic supplementary material:**

The online version of this article (10.1245/s10434-019-07741-w) contains supplementary material, which is available to authorized users.

Breast cancer, the prevailing cancer type for females worldwide, affects women across all ages and ethnicities, making it a necessity to continuously advance treatment and diagnosis regimes. In the past year, the global incidence of breast cancer counted 2.1 million newly diagnosed cases with mortality reaching 40,000 patients per year in the United States and 138,000 in Europe alone.^1^–^5^

Evaluating the response to chemotherapy with the goal to improve disease-free and overall survival has become an important milestone in monitoring the treatment of breast cancer patients and for clinical research.^6^–^8^ Neoadjuvant chemotherapy, a well-established method of treatment for a select group of patients, allows radiographic, clinical, and pathological monitoring of tumor response before to the definite operation.^9^–^15^ One of the main purposes of neoadjuvant treatment modalities is to observe directly its effects on tumor response in a relatively short timeframe, allowing the subsequent pathological response evaluation. In fact, pathological assessment of the surgical specimen is considered the most accurate method for chemotherapy evaluation.^8^,^12^,^16^ Previous studies have shown that pathological complete remission (pCR) strongly correlates with improved overall (OS) and recurrence-free survival (RFS).^16^–^23^ Although pCR, due to this correlation, has been considered the most important surrogate end point for clinical trials, the prognostic value depends on its definition, which varies depending on the used evaluation method.^13^,^19^,^24^,^25^ Current recommendations suggest the use of a histological classification for the assessment of the pathological response but do not favor a particular grading system.^26^ For international clinical trials, systems that include pretreatment cellularity, such as the Miller-Payne or the Pinder system, have been recommended.^20^,^26^ Additional response classifications have been established and used, such as Chevallier and Sataloff score, and more recently the residual cancer burden (RCB) score. As one of the developed scores for the evaluation of pCR, the RCB has demonstrated reliable reproducibility, a wide range of significant prognostic pathological elements, and prognostic power across various breast cancer subtypes and chemotherapy regimens.^24^,^27^–^32^ There are several advantages of the RCB score compared to other methods. The RCB score is based on the mathematical model and, as opposite to other methods segregating response into groups, RCB is an exact deliverable with a precisely calculated score. Its main advantage is the potential to identify a patient subgroup with an excellent prognosis despite (minimal) residual disease (RCB I).

So far, the RCB score has only been used to a limited extent by independent researchers outside of the MD Anderson Cancer Center.^15^,^27^,^33^–^36^ In the following study, we investigated the prognostic impact of the RCB score with special emphasis on RFS in a breast cancer collective treated at a single oncology unit and analyzed at a single pathology department.

## Methods

### Study Design

We performed a retrospective analysis of 184 patients with early or locally advanced breast cancer, who underwent neoadjuvant therapy at the Division of Oncology, Department of Internal Medicine, University Hospital, Medical University of Graz, Austria, before undergoing surgical treatment in curative intent at three departments of surgery all related to our local oncology network (Hospital Graz Sued-West, Brothers of Charity Hospital and Elisabethinen Hospital Graz, Austria). These 184 patients were drawn from 213 patients, who had undergone surgery at these three surgical departments after neoadjuvant therapy between January 1, 2011 and December 31, 2016. We excluded 29 patients who (1) underwent neoadjuvant endocrine therapy (*n* = 23), (2) progressed during neoadjuvant therapy (*n* = 3), (3) had sentinel lymph node excision before neoadjuvant therapy (*n* = 2), and (4) did not have available follow-up data (*n* = 1). For all patients, the pathology procedure and diagnosis was performed at the Department of Pathology, Hospital Graz Sued-West, including prospective assessment of the RCB score and class and estrogen and progesterone receptor, HER2 (human epidermal growth factor receptor 2), and Ki67 labeling index (Ki67 LI). We retrospectively ascertained demographic variables, clinicopathological data from the biopsy prior neoadjuvant therapy, the postoperative pathology report, and follow-up data after surgery (local recurrence, distant metastasis, second mammary, and nonmammary tumors, death) via investigation of the local electronic healthcare databases, paper charts, and telephone interviews. All patients underwent a structured postsurgical follow-up, with regular clinical visits and selected imaging studies every 3 months during the first 3 years, every 6 months during the subsequent 2 years, and yearly thereafter for a maximum duration of 10 years.

The date of definitive surgery was defined as the baseline data. Primary endpoint of this study was 5-year RFS, defined as a composite of local recurrence from breast cancer, distant metastasis from breast cancer, or death-from-any-cause, whatever came first, during the first 5 years of follow-up. The primary endpoint was ascertained retrospectively and was adjudicated by two oncologists (HDM & MB). Metachronous contralateral breast cancer occurring during follow-up was not counted as primary endpoint event. The secondary endpoint was 5-year OS, defined as the time between surgery and death from any cause or censoring alive, respectively.

## Pathology

### Pretherapeutic Procedure

Before therapy, all cases were histologically diagnosed by core needle biopsy on which also ER, PR, HER2, and Ki67 LI were analyzed by immunohistochemistry. For the assessment of ER and PR the Allred score, for HER2 the 2013 ASCO/CAP guidelines were used.^25^–^27^,^37^ Equivocal results for HER2 on immunohistochemistry were further evaluated by in situ hybridization using DDISH (Roche-Ventana^®^, Ventana, CA). The Ki67 LI was assessed by estimation using 10% steps except for the area between 10% and 20% for which 5% steps were used.^38^ If a distinction between 10% steps was not possible, a range for Ki67 LI (e.g., 20–30%) was included in the pathological report. Immunohistochemistry and in situ hybridization were performed on an automated stainer (BenchMark Ultra™, Roche-Ventana^®^). Histological typing and grading were performed according to WHO.^39^ The carcinomas also were categorized in molecular subgroups based on the four immunohistochemical analyses.^37^,^40^,^41^ When luminal tumors were diagnosed, only selected tumors with either higher stage or luminal B-like tumors with higher proliferation were selected for neoadjuvant chemotherapy.

### Postoperative Procedure

All surgical specimens were intraoperatively submitted to the pathology department without prior fixation. The margins were inked, the specimens sliced at 4–5 mm, and subsequently assessed grossly. If required by close distance of the tumor from the margins, they also were assessed by frozen section. After fixation in 4% buffered formalin for at least 12 h, specimens after breast-conserving therapy were totally embedded in serial order from the mammillary to the peripheral end using either usual or gross cassettes. From mastectomy specimens, the tumor bed was usually subtotally embedded. If no residual tumor was grossly visible, the tumor bed was completely embedded. The pathology report included the histopathological tumor type and grade, status of the surgical margins, ypTNM classification, including vascular and lymph vascular invasion, and the regression grading using the RCB score. The RCB score was assessed by using the RCB calculator on the MD Anderson website and reported including the particular class.^17^,^27^,^42^ If residual tumor was present, the analysis for ER, PR, HER2, and Ki67 LI was repeated.

### Statistical Methods

All statistical analyses were performed with Stata^®^ 15.0 by FP (Stata Corp., Houston, TX). Continuous variables were reported as medians [25th–75th percentile] and count data as absolute numbers (%). The distribution of continuous variables between two groups was compared with rank sum-tests, whereas the association between two categorical variables was investigated with chi^2^ (*χ*^2^) and Fisher’s exact tests. Simple and multiple linear regression models were fitted to examine the association between covariates and the continuous RCB score. Median follow-up time was estimated with the reverse Kaplan–Meier estimation.^43^ RFS and OS were computed with Kaplan–Meier estimators, and RFS and OS functions were compared between two or more groups using log-rank tests. The association between prognostic variables, such as the RCB scores and classes, respectively, and the hazards of recurrence or death were calculated using uni- and multivariable Cox proportional hazards models. The RCB was investigated as (1) a continuous variable (RCB “index” from 0 to 4), (2) a binary variable with an empirical prespecified cutoff at the 75th percentile of the RCB distribution (in the absence of validated cutoffs for the RCB “index”), and (3) as a 4-level categorical variable (RCB “classes” 0, 1, 2, and 3). The proportionality of hazards assumption was assessed by fitting interactions between the continuous RCB index and linear follow-up time. The discriminative performance of the RCB indices and classes, respectively, toward 5-year RFS were quantified with Harell’s c-index.^44^ Potential effect modifiers (“interactions”) of RCB with clinically relevant subgroup variables were investigated by fitting interactions between the RCB score and classes, respectively, and selected covariates within a Cox model.

## Results

### Analysis of the Neoadjuvant Treatment Phase and Definitive Surgery

A total of 184 female patients were included in the analysis. The median age of the cohort at diagnosis was 54 years [25th–75th percentile: 47–64] (Table 1). Histologically, the majority of the 184 invasive carcinomas were of no special type and grade 3 (*n* = 122, 66%) with a median Ki67 LI of 35% [15–60]. One third (*n* = 61, 33%) of the carcinomas were HER2 amplified, and 49 carcinomas (27%) were triple-negative. Ten (5%) patients had been previously diagnosed with breast cancer. After diagnosis, 169 (92%) patients were treated with sequential anthracycline-taxane neoadjuvant chemotherapy and the remaining 15 (8%) with other neoadjuvant chemotherapy regimens. HER2 directed therapy was given as indicated (Table 1). All analyses were performed in the entire cohort (*n* = 184) and in the group of patients treated with anthracycline and taxane neoadjuvant chemotherapy (*n* = 169), and there were no differences in the main findings. Therefore, we only show data for the entire cohort. Primary definitive surgery was breast-conserving therapy in 118 (64%) and mastectomy in 66 (36%) patients. Axillary lymph-node dissection and sentinel node biopsy were performed after neoadjuvant therapy in 157 patients (85%) and 27 (15%) patients, respectively.

**Table 1:** Baseline characteristics of the study population (*n* = 184)

Variable	*n* (% miss.)^†^	Overall (*n* = 184)	No recurrence or death during follow-up (*n* = 141)	Recurrence or death during follow-up (*n* = 43)	*p*^‡^
Demographic characteristics					
Age at entry (years)	184 (0)	54 [47–64]	54 [47–63]	56 [49–64]	0.425
Female Gender	184 (0)	184 (100%)	141 (100%)	43 (100%)	N/A
Characteristics at diagnosis					
Molecular breast cancer subtype	184 (0)	–	–	–	0.418
ER+/PR+ or –/HER2−	–	74 (40%)	53 (38%)	21 (49%)	–
HER2+ (classical and variants)	–	61 (33%)	49 (35%)	12 (28%)	–
Triple-negative	–	49 (27%)	39 (28%)	10 (23%)	–
Tumor grade	184 (0)	–	–	–	0.472
G1	–	3 (2%)	3 (2%)	0 (0%)	–
G2	–	59 (32%)	47 (33%)	12 (28%)	–
G3	–	122 (66%)	91 (65%)	31 (72%)	–
Ki67 labeling index (%)	184 (0)	35 [15–60]	35 [15–60]	40 [20–50]	0.724
Menopausal status	184 (0)	–	–	–	0.443
Premenopausal/perimenopausal	–	73 (40%)	58 (41%)	15 (35%)	–
Postmenopausal	–	102 (55%)	75 (53%)	27 (63%)	–
Unclear/unknown/missing	–	9 (5%)	8 (6%)	1 (2%)	–
Neoadjuvant and surgical treatment characteristics and outcomes					
Neoadjuvant systemic therapy	184 (0)	–	–	–	**0.026**
Anthracycline + Taxane (± anti-Her2)	–	169 (92%)	133 (94%)	36 (84%)	–
Other regimens (± anti-Her2)^§^	–	15 (8%)	8 (6%)	7 (16%)	–
Surgical outcome	184 (0)	–	–	–	**0.017**
#NAME?	–	118 (64%)	97 (69%)	21 (49%)	–
Ablation	–	66 (36%)	44 (31%)	22 (51%)	–
Definitive axillary procedure	184 (0)	–	–	–	**0.009**
Sentinel node biopsy (SNB)	–	27 (15%)	26 (18%)	1 (2%)	–
Axillary lymph node dissection (ALND)	–	157 (85%)	115 (82%)	42 (98%)	–
Post-neoadjuvant tumor category (ypT)	184 (0)	–	–	–	**< 0.0001**
ypTis-ypT0	–	56 (30%)	40 (35%)	7 (16%)	–
ypT1	–	87 (47%)	72 (51%)	15 (35%)	–
ypT2	–	26 (14%)	14 (10%)	12 (28%)	–
ypT3-ypT4	–	15 (8%)	6 (4%)	9 (21%)	–
Number of positive nodes (pN)	184 (0)	–	–	–	**< 0.0001**
Post-neoadjuvant nodal status (TNM ypN)	184 (0)	–	–	–	**< 0.0001**
ypN0	–	135 (73%)	113 (80%)	22 (51%)	–
ypN1	–	27 (15%)	19 (13%)	8 (19%)	–
ypN2	–	13 (7%)	7 (5%)	6 (14%)	–
ypN3	–	9 (5%)	2 (1%)	7 (16%)	–
Adjuvant endocrine therapy	184 (0)	92 (50%)	72 (51%)	20 (47%)	0.601
Residual cancer burden (RCB) variables					
RCB score	184 (0)	1.57 [0–2.41]	1.39 [0–1.87]	2.34 [1.28–3.96]	**< 0.0001**
RCB class	184 (0)	–	–	–	**< 0.0001**
0	–	51 (28%)	45 (32%)	6 (14%)	–
1	–	29 (16%)	24 (17%)	5 (12%)	–
2	–	72 (39%)	59 (42%)	13 (30%)	–
3	–	32 (17%)	13 (9%)	19 (44%)	–

Distribution overall as well as by recurrence-free survival event status. Data are reported as medians [25th–75th percentile] for continuous variables, and as absolute frequencies (%) for count data, respectively

*N/A* not applicable, *RCB* residual cancer burden, *ER* estrogen receptor, *PR* progesterone receptor; or –/*HER*-*2* human epidermal growth factor receptor 2 (erb-B2); *TNM* tumor node metastasis classification

^†^Number of patients with fully observed data (% missing)

^‡^*p* values were derived using Wilcoxon’s rank-sum tests, *χ*^2^ tests, or Fisher’s exact tests (*p* ≤ 0.05 are reported in bold font)

^§^Other regimens include mostly patients who received only anthracyclines or only taxanes, mainly related to toxicity

### Analysis of RCB

The median RCB score was 1.57 with a range from 0 to 4.94. Corresponding RCB classes are reported in Table 1, and outcomes by intrinsic breast cancer subtype in Supporting Table 1. In linear regression, the strongest uni-variable predictors of a low RCB score were triple-negative and HER2-positive subtypes and higher Ki67 LI, whereas higher age predicted for a higher RCB score (Table 2). In a multivariable regression model, only breast cancer subtypes and Ki67 LI prevailed as independent predictors of RCB (Supporting Table 2).

**Table 2:** Simple linear regression models investigating predictors of the RCB score (*n* = 184)

Variable	Regression coefficient *β*	95% CI	*p*
Age at entry (per 5 years increase)	0.12	0.04–0.21	**0.004**
Molecular breast cancer subtype	/	/	/
ER+/PR+ or –/HER2−	Ref.	Ref.	Ref.
HER2+ (classical and variants)	− 1.27	− 1.70 to (− 0.85)	**< 0.0001**
Triple-negative	− 1.02	− 1.47 to (− 0.57)	**< 0.0001**
Tumor grade G3	−0.51	− 0.92 to (− 0.09)	**0.017**
Ki-67 labeling index (per 10% increase)	− 0.18	− 0.25 to (− 0.11)	**< 0.0001**
Postmenopausal status	0.39	− 0.01 to 0.78	0.053
Neoadjuvant systemic therapy—other regimens than sequential anthracycline + taxane (± anti-Her2)	0.76	0.05 to 1.48	**0.037**

*ER* estrogen receptor, *PR* progesterone receptor; or –/*HER*-*2* human epidermal growth factor receptor 2 (erb-B2)

### Analysis of the Primary Endpoint: Recurrence-free Survival (RFS)

Median follow-up (with follow-up truncated at 5 years) was 4 years; 75% of patients were followed for at least 3 years. During this interval, we observed 38 recurrences, and 28 patients died (*n* = 43 RFS events). The 1-, 2-, 3-, and 5-year RFS estimates were 93%, 86%, 80%, and 73%, respectively (Supporting Figure 1). The observed recurrences (*n* = 38) included local recurrence (*n* = 10; including skin and lymph nodes), distant lymph node metastasis (*n* = 1), visceral metastases (*n* = 17; cerebral, liver, gastrointestinal), and nonvisceral metastases (*n* = 10; bone, lung, pleural). In addition, we observed contralateral breast cancer in five patients and deaths from other causes, including other malignancies in three patients, which were not included in the analysis.

Average RCB scores were significantly higher in the 43 patients who developed recurrence and/or died during follow-up compared with the 141 who remained event-free (median index: 2.34 vs. 1.39 points, rank-sum *p* < 0.0001). In a uni-variable Cox regression, higher RCB scores were associated with worse RFS (hazard ratio (HR) per 1 point increase = 1.80, 95% CI 1.44–2.24, *p* < 0.0001, Table 3).

**Table 3:** Predictors of 5-year recurrence-free survival (RFS) univariable Cox regression

Variable	HR	95% CI	*p*
RCB score (by 1 point increase)	1.8	1.44–2.24	**< 0.0001**
RCB class	–	–	–
0	Ref.	Ref.	Ref.
1	1.43	0.44–4.68	0.556
2	1.45	0.54–3.87	0.456
3	6.66	2.66–16.70	**< 0.0001**
Age at entry (per 5 years increase)	1.04	0.91–1.18	0.61
Molecular breast cancer subtype	–	–	–
ER+/PR+ or –/HER2−	Ref.	Ref.	Ref.
HER2+ (classical and variants)	0.75	0.37–1.54	0.437
Triple-negative	0.82	0.38–1.74	0.6
Tumor grade G3	1.56	0.78–3.10	0.206
Ki-67 labeling index (per 10% increase)	1.02	0.92–1.14	0.67
Postmenopausal status	1.35	0.72–2.51	0.35
Neoadjuvant systemic therapy—other regimens than sequential anthracycline + taxane (± anti-Her2)^†^	2	0.84–4.74	0.118
Breast conservation	0.53	0.29–0.97	**0.038**
Axillary lymph node dissection (ALND)	7.36	1.01–53.56	**0.049**
Post-neoadjuvant tumor size (TNM ypT)	–	–	–
ypTis-ypT0	Ref.	Ref.	Ref.
ypT1	1.38	0.56–3.39	0.479
ypT2	4.29	1.69–10.91	**0.002**
ypT3-ypT4	5.78	2.09–15.95	**0.001**
Number of positive nodes (per 1 increase)	1.13	1.08–1.18	**< 0.0001**
Post-neoadjuvant nodal status (TNM ypN)	–	–	–
ypN0	Ref.	Ref.	Ref.
ypN1	1.99	0.88–4.51	0.097
ypN2	3.77	1.52–9.35	**0.004**
ypN3	7.38	3.11–17.51	**< 0.0001**
Adjuvant endocrine therapy	0.75	0.41–1.37	0.348

*HR* hazard ratio, *CI* confidence interval, *Ref*. Reference group, *RCB* residual cancer burden, *ER* estrogen receptor, *PR* progesterone receptor; or –/*HER*-*2* human epidermal growth factor receptor 2 (erb-B2), *N/E* not estimable because no patients in the sentinel node biopsy group developed recurrence or died, *TNM* tumor node metastasis classification

^†^Other regimens include mostly patients who received only anthracyclines or only taxanes, mainly related to toxicity

**Fig. 1:**
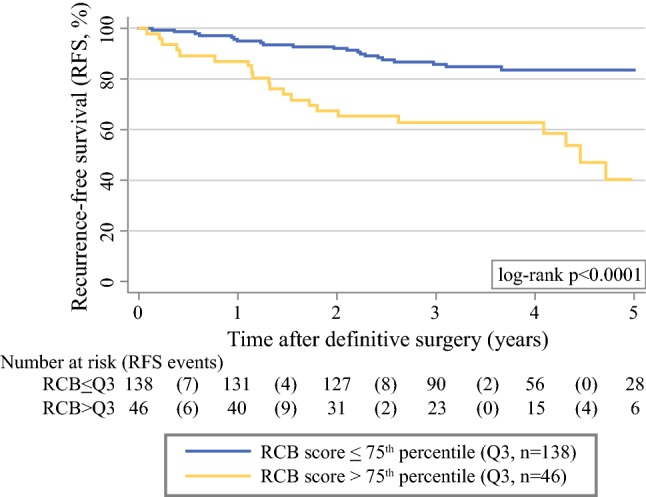
Kaplan–Meier recurrence-free survival (RFS) functions by RCB score. Patients were dichotomized into two groups based on having an RCB score > (*n* = 46) or ≤ (*n* = 138) the 75th percentile (Q3) of its distribution. The Q3 cutoff is at 2.41 RCB index points. Numbers below the Kaplan–Meier plot represent a risk table, with the number of RFS events occurring within the respective interval report in round brackets

Five-year RFS estimates were 40% (95% CI 20–59) in the 46 patients with an RCB index above the 75th percentile of its distribution (cutoff: 2.41 points), and 84% (95% CI 76–89) in the 138 patients with an RCB score equal or below this cutoff (log-rank *p* < 0.0001, Fig. 1).

**Fig. 2:**
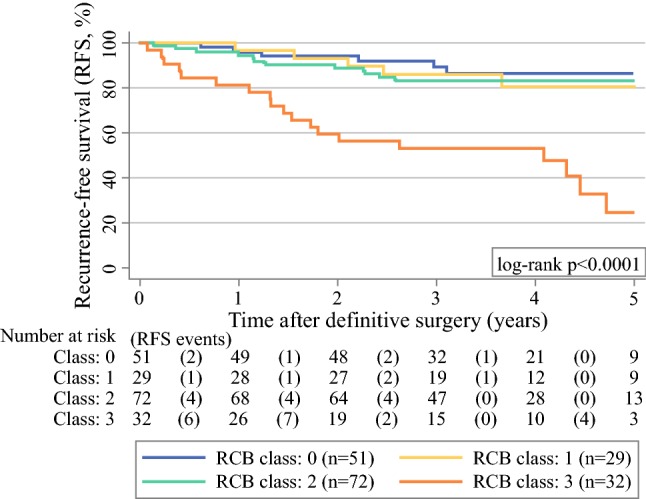
Kaplan–Meier recurrence-free survival (RFS) function by RCB class. Numbers below the Kaplan–Meier plot represent a risk table per RCB class, with the number of RFS events occurring within the respective interval report in round brackets

The RCB class was also strongly associated with RFS in univariable Cox regression (Table 3), but the RFS experience of patients with RCB classes 0–2 did not appear to differ substantially at 5 years (Fig. 2). The strongest other univariable predictors of RFS were residual tumor and residual axillary lymph node metastases as determined by the ypTNM categories, which are by definition components of the RCB (Table 3).

### Analysis of RCB’s Predictive Performance, Time-Dependent Impact on RFS, and Potential Subgroup Effects

The continuous RCB score achieved a Harell’s c-index of 0.70 for discriminating between patients who experienced the primary endpoint during follow-up or not. The corresponding c-index of the RCB class variable was slightly lower (Harell’s c = 0.67). Importantly, we did not observe evidence for non-proportionality of hazards according to the RCB score (interaction HR with linear follow-up time = 1.00, *p* = 0.896). This is consistent with the hypothesis that the adverse prognostic impact of the RCB score on RFS prevails throughout follow-up period. Indeed, HRs for RFS per 1 point increase in the RCB score was 1.83 (95% CI 1.22–2.74, *p* = 0.003) for the first year after definitive surgery, 1.72 (95% CI 1.35–2.18, *p* < 0.0001) for the first 3 years after surgery, and 1.80 (95% CI 1.44–2.24, *p* < 0.0001) for the first 5 years after surgery, respectively. Finally, in an exploratory hypothesis-generating analysis, we investigated whether the adverse impact of RCB on RFS may be different across selected clinically important subgroups. We found that the association between higher RCB score and worse RFS was consistent across different molecular subtypes, as well as for tumor grade (Supporting Table 3). However, the adverse prognostic impact of RCB as indicated by the RCB score on RFS was more pronounced in patients with a high Ki67 LI, in the elderly, as well as in those that could not receive sequential anthracycline-taxane based chemotherapy.

#### Analysis of the Secondary Endpoint: Overall Survival

**Fig. 3:**
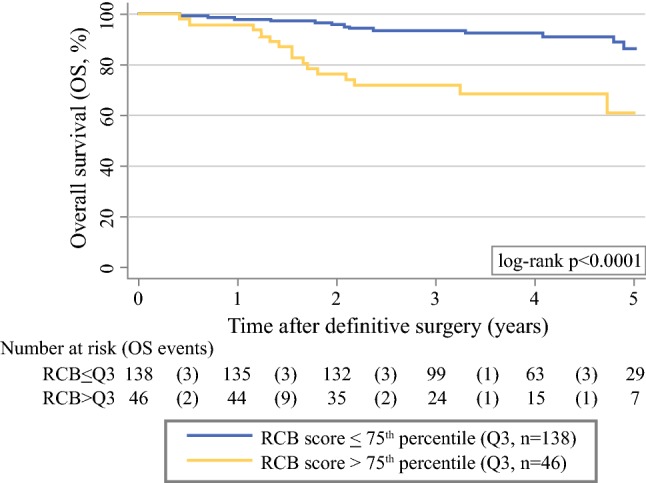
Kaplan–Meier overall survival (OS) function by RCB score. Patients were dichotomized into two groups based on having an RCB score > (*n* = 46) or ≤ (*n* = 138) the 75th percentile (Q3) of its distribution. The Q3 cutoff is at 2.41 RCB score points. Numbers below the Kaplan–Meier plot represent a risk table, with the number of OS events occurring within the respective interval report in round brackets

The 1-, 2-, 3-, and 5-year overall survival (OS) estimates were 97%, 91%, 88%, and 80%, respectively (Supporting Figure 2). Five-year OS estimates were 61% (95% CI 10–40) in the 46 patients with an RCB score > 3rd quartile and 86% (95% CI 76–92) in the 138 patients with an RCB score ≤ 3rd quartile (log-rank *p* < 0.0001; Fig. 3). Similar to the RFS analysis, the adverse impact of the RCB class on OS was driven by class 3 patients, whereas survival outcomes for class 0–2 patients were relatively similar (Supporting Figure 3). In Cox regression, both higher RCB scores and classes emerged as prognostic factors for worse OS (Supporting Table 4).

## Discussion

In this study, we retrospectively evaluated the feasibility of the RCB scoring and classification and its impact on prognosis of patients treated with neoadjuvant systemic therapy. Because neoadjuvant systemic therapy has significantly improved survival and is the approach of choice in the selected high-risk population of localized primary breast cancer, this analysis is of great importance in the real-world clinical setting. Our cohort shows a mixture of plausible incidence of high risk breast cancer patients (Table 1: TNBC 27% and HER2-positive 33%), offering comparable distributions of these molecular subtypes to the RCB validation cohorts (HER2 pos. 1–53%, triple-negative breast cancer 21–29%) published by Symmans et al.^17^,^27^,^32^ Although our study showed some limitations, we found that the prognostic impact of RCB was consistent over time and an even distribution of hazards made it to an extent universally applicable.

Several differences to the original validation cohort need to be addressed. Patients who received less than 75% of the prescribed cycles of chemotherapy, as well as patients with previous invasive breast cancer and possible genetic predisposition for reoccurring breast cancer, were included in our study. Furthermore, we did not differentiate according to chemotherapy administered, because most patients had received an anthracycline- and taxane-based chemotherapy. Presently, recurrences are only roughly categorized, because a broad spectrum of different types of events was observed in our relatively small cohort. By extending the population cohort, we will be able to achieve a better categorization of the recurrences in future. However, we strongly believe that the prognostic impact of RCB would thereby remain unchanged. In addition, because in the original study by Symmans et al.^27^ clinical (c-) stage and age were not independent prognosticators for the overall cohort, in relation to RCB, we decided not to analyze them separately. Moreover, we chose a 5-year primary endpoint, resulting in a shorter follow-up period compared with the original validation cohort, although notably we used the date of surgery and not the date of the initial histological diagnosis to calculate RFS.^27^ This shorter follow-up might limit our results and, in particular, may lead to a falsely improved disease-free survival for luminal subtypes, because these recurrences tend to occur later.^45^ However, it has been shown that pCR and also RCB play a greater role in aggressive breast cancer subtypes.^29^,^31^ Therefore, despite these limitations of the study, we were able to show stable results with only little variation to Symmans cohort concerning the prognostic impact of RCB.^23^,^24^

The strength of this study is undoubtedly the consistency regarding the systemic oncological treatment, including the pathological processing, which could only be achieved through the comparatively small cohort. All patients were treated within the Division of Oncology, Department of Internal Medicine at the University Hospital under the same protocols and standards of treatment. All surgical specimens were processed in the Department of Pathology in Hospital Graz Sued-West using a standardized protocol for grossing and embedding and standardized cancer reports, including RCB, which was assessed prospectively according to the protocol and the website developed by Symmans et al.^17^,^27^,^42^

In our study, we were able to demonstrate that nodal status after chemotherapy, tumor size, and number of positive lymph nodes at diagnosis showed, in accordance with other research, a strong impact on RFS and OS.^29^,^30^,^41^ Because all of these factors are included in the calculation of the RCB, their individual impact has positively influenced the prognostic value of RCB on RFS and OS.^27^ Furthermore, we were able to show that the prognostic difference between the low RCB classes is small and does not reach statistical significance. This finding suggests that the prognostic impact of RCB on RFS is mainly driven by the highest RCB class, which has been previously observed by Symmans et al.^27^ for the HER2-amplified population, in which the prognostic impact between RCB classes I and II was not significant. However, our cohort was relatively small, and it can be reasonably speculated that a larger sample size with a longer follow-up and consequently higher event rate would have rendered the small numerical differences in survival outcomes between patients with RCB class 0–1 and RCB class 2 statistically significant. Otherwise, the adverse impact of higher RCB scores on RFS was consistent across several clinical subgroups but more pronounced in patients with high Ki67 LI, suggesting that residual disease from highly proliferative tumors may be more detrimental than residual masses from slowly proliferating tumors. This finding concurs with other research.^46^,^47^

We were able to show that the strongest uni-variable predictors of a lower RCB score were high risk molecular subtypes of breast cancer (triple-negative breast cancer, HER2-amplified, and breast cancer with high Ki67), which in general tend to have a greater risk for recurrence but also higher rates of pCR in response to neoadjuvant chemotherapy.^45^ This could be explained by the fact that these more aggressive molecular subtypes are more vulnerable to cytotoxic treatment and can be targeted by specific drugs.^48^,^49^ In these breast cancer subtypes, neoadjuvant chemotherapy and targeted treatment leads primarily to a better response, which is better reflected by RCB compared to the pCR rate.^7^ For these more aggressive molecular subtypes, pCR at surgery is particularly associated with longer RFS and OS, even comparable to less aggressive molecular breast cancer subtypes.^7^ In addition, incomplete pathological response with similar prognostic impact as pCR can be easily measured and determined using RCB.^31^

Concerning less aggressive molecular subtypes, higher age confounds with statistically higher probability for hormone receptor-positive breast cancer, which generally has a better overall prognosis but benefits less from taxane-based neoadjuvant chemotherapy.^5^ The combination of patients’ higher age and a high RCB score is associated with worse prognosis (Table 3 and Supporting Table 2). This observation may help facilitate the decision toward omitting neoadjuvant chemotherapy in older patients with hormone receptor positive disease, knowing that they have worse prognosis. Thus, these patients could be selected for novel neoadjuvant approaches.

Our analysis provides a scientific confirmation of the RCB and an excellent basis for the risk stratification of patients in daily routine practice, both in the follow-up and in selection of patients for novel systemic approaches and clinical studies in the future. The risk stratification based on the RCB provides a better tool to identify patients who need less attention in the follow-up but also selects patients with dismal prognosis who are candidates for postneoadjuvant clinical studies. We were able to show that RCB can be used in routine clinical setting, where it demonstrates valuable prognostic impact for disease-specific outcome in breast cancer patients following neoadjuvant therapy. Our findings reveal reliable results concerning the prognostic impact compared to the original investigators from MD Anderson. Although our cohort showed the RCB as highly significant, stable, prognostic parameter, it cannot serve as confirmation for long-term prognosis, particularly for patients with hormone receptor-positive disease. Therefore, it is warranted to further investigate the potential of RCB for guiding the intensity and duration of follow-up or patient selection for clinical trials.

## Electronic supplementary material

Below is the link to the electronic supplementary material.

Supplementary material 1 (PDF 120 kb)

Supplementary material 2 (PDF 117 kb)

Supplementary material 3 (PDF 171 kb)

Supplementary material 4 (PDF 34 kb)

Supplementary material 5 (PDF 234 kb)

Supplementary material 6 (PDF 100 kb)

Supplementary material 7 (PDF 97 kb)

## Data Sharing and Data Accessibility

All data generated or analyzed during this study are included in this published article (and its supporting information files). Statistical analysis code is available on request from FP. The dataset analyzed during the current study cannot be shared under the current ethics committee approval.

## References

[1] Akram M, Iqbal M, Daniyal M, Khan AU (2017). Awareness and current knowledge of breast cancer. Biol Res.

[2] Ferlay J, Colombet M, Soerjomataram I (2018). Cancer incidence and mortality patterns in Europe: estimates for 40 countries and 25 major cancers in 2018. Eur J Cancer.

[3] DeSantis CE, Ma J, Goding Sauer A, Newman LA, Jemal A (2017). Breast cancer statistics, 2017, racial disparity in mortality by state. CA Cancer J Clin.

[4] DeSantis C, Ma J, Bryan L, Jemal A (2014). Breast cancer statistics, 2013. CA Cancer J Clin.

[5] Bray F, Ferlay J, Soerjomataram I, Siegel RL, Torre LA, Jemal A (2018). Global cancer statistics 2018: GLOBOCAN estimates of incidence and mortality worldwide for 36 cancers in 185 countries. CA Cancer J Clin.

[6] Bray F, McCarron P, Parkin DM (2004). The changing global patterns of female breast cancer incidence and mortality. Breast Cancer Res.

[7] Berruti A, Amoroso V, Gallo F (2014). Pathologic complete response as a potential surrogate for the clinical outcome in patients with breast cancer after neoadjuvant therapy: a meta-regression of 29 randomized prospective studies. J Clin Oncol.

[8] Von Minckwitz G, Untch M, Blohmer J-U (2012). Definition and impact of pathologic complete response on prognosis after neoadjuvant chemotherapy in various intrinsic breast cancer subtypes. J Clin Oncol.

[9] Chen JH, Bahri S, Mehta RS (2014). Impact of factors affecting the residual tumor size diagnosed by MRI following neoadjuvant chemotherapy in comparison to pathology. J Surg Oncol.

[10] Cardoso F, Harbeck N, Fallowfield L, Kyriakides S, Senkus E, ESMO Guidelines Working Group (2012). Locally recurrent or metastatic breast cancer: ESMO Clinical Practice Guidelines for diagnosis, treatment and follow-up. Ann Oncol.

[11] Schott AF, Roubidoux MA, Helvie MA (2005). Clinical and radiologic assessments to predict breast cancer pathologic complete response to neoadjuvant chemotherapy. Breast Cancer Res Treat.

[12] Schott AF, Hayes DF (2012). Defining the benefits of neoadjuvant chemotherapy for breast cancer. J Clin Oncol.

[13] Mazouni C, Peintinger F, Wan-Kau S (2007). Residual ductal carcinoma in situ in patients with complete eradication of invasive breast cancer after neoadjuvant chemotherapy does not adversely affect patient outcome. J Clin Oncol.

[14] Honkoop A, Van Diest P, De Jong J (1998). Prognostic role of clinical, pathological and biological characteristics in patients with locally advanced breast cancer. Br J Cancer.

[15] Cockburn A, Yan J, Rahardja D (2014). Modulatory effect of neoadjuvant chemotherapy on biomarkers expression; assessment by digital image analysis and relationship to residual cancer burden in patients with invasive breast cancer. Hum Pathol.

[16] Fisher B, Bryant J, Wolmark N (1998). Effect of preoperative chemotherapy on the outcome of women with operable breast cancer. J Clin Oncol.

[17] Symmans WF, Peintinger F, Hatzis C (2007). Measurement of residual breast cancer burden to predict survival after neoadjuvant chemotherapy. J Clin Oncol..

[18] Rouzier R, Extra J-M, Klijanienko J (2002). Incidence and prognostic significance of complete axillary downstaging after primary chemotherapy in breast cancer patients with T1 to T3 tumors and cytologically proven axillary metastatic lymph nodes. J Clin Oncol.

[19] Earl H, Provenzano E, Abraham J (2015). Neoadjuvant trials in early breast cancer: pathological response at surgery and correlation to longer term outcomes–what does it all mean?. BMC Med.

[20] Romero A, Garcia-Saenz J, Fuentes-Ferrer M (2012). Correlation between response to neoadjuvant chemotherapy and survival in locally advanced breast cancer patients. Ann Oncol.

[21] Hennessy BT, Hortobagyi GN, Rouzier R (2005). Outcome after pathologic complete eradication of cytologically proven breast cancer axillary node metastases following primary chemotherapy. J Clin Oncol.

[22] Guarneri V, Broglio K, Kau S-W (2006). Prognostic value of pathologic complete response after primary chemotherapy in relation to hormone receptor status and other factors. J Clin Oncol.

[23] Abrial SC, Penault-Llorca F, Delva R (2005). High prognostic significance of residual disease after neoadjuvant chemotherapy: a retrospective study in 710 patients with operable breast cancer. Breast Ccancer Res Treat.

[24] Provenzano E, Bossuyt V, Viale G (2015). Standardization of pathologic evaluation and reporting of postneoadjuvant specimens in clinical trials of breast cancer: recommendations from an international working group. Mod Pathol.

[25] Kaufmann M, Hortobagyi GN, Goldhirsch A (2006). Recommendations from an international expert panel on the use of neoadjuvant (primary) systemic treatment of operable breast cancer: an update. J Clin Oncol.

[26] Bossuyt V, Provenzano E, Symmans W (2015). Recommendations for standardized pathological characterization of residual disease for neoadjuvant clinical trials of breast cancer by the BIG-NABCG collaboration. Ann Oncol.

[27] Symmans WF, Wei C, Gould R (2017). Long-term prognostic risk after neoadjuvant chemotherapy associated with residual cancer burden and breast cancer subtype. J Clin Oncol.

[28] Naidoo K, Parham DM, Pinder SE (2017). An audit of residual cancer burden reproducibility in a UK context. Histopathology.

[29] Choi M, Park YH, Ahn JS (2016). Assessment of pathologic response and long-term outcome in locally advanced breast cancers after neoadjuvant chemotherapy: comparison of pathologic classification systems. Breast Cancer Res Treat.

[30] Corben AD, Abi-Raad R, Popa I (2013). Pathologic response and long-term follow-up in breast cancer patients treated with neoadjuvant chemotherapy: a comparison between classifications and their practical application. Arch Pathol Lab Med.

[31] Campbell JI, Yau C, Krass P (2017). Comparison of residual cancer burden, American Joint Committee on Cancer staging and pathologic complete response in breast cancer after neoadjuvant chemotherapy: results from the I-SPY 1 TRIAL (CALGB 150007/150012; ACRIN 6657). Breast Cancer Res Treat.

[32] Peintinger F, Sinn B, Hatzis C (2015). Reproducibility of residual cancer burden for prognostic assessment of breast cancer after neoadjuvant chemotherapy. Mod Pathol.

[33] Sharma P, López-Tarruella S, García-Saenz JA (2018). Pathological response and survival in triple-negative breast cancer following neoadjuvant carboplatin plus docetaxel. Clin Cancer Res.

[34] Quintela-Fandino M, Lluch A, Manso L (2017). 18F-fluoromisonidazole PET and activity of neoadjuvant nintedanib in early HER2-negative breast cancer: a window-of-opportunity randomized trial. Clin Cancer Res.

[35] Cottu P, D’hondt V, Dureau S (2018). Letrozole and palbociclib versus chemotherapy as neoadjuvant therapy of high-risk luminal breast cancer. Ann Oncol..

[36] Martín M, Chacón JI, Antón A (2017). Neoadjuvant therapy with weekly nanoparticle albumin-bound paclitaxel for luminal early breast cancer patients: results from the NABRAX Study (GEICAM/2011-02), a multicenter, non-randomized, phase II Trial, with a companion biomarker analysis. Oncologist.

[37] Hammond MEH, Hayes DF, Dowsett M (2010). American Society of Clinical Oncology/College of American Pathologists guideline recommendations for immunohistochemical testing of estrogen and progesterone receptors in breast cancer (unabridged version). Arch Pathol Lab Med.

[38] Kosa C, Kardos L, Kovacs J, Szollosi Z (2013). Comparison of dual-color dual-hapten brightfield in situ hybridization (DDISH) and fluorescence in situ hybridization in breast cancer HER2 assessment. Pathol Res Pract.

[39] Sinn H-P, Kreipe H (2013). A brief overview of the WHO classification of breast tumors. Breast Care.

[40] Wolff AC, Hammond MEH, Schwartz JN (2007). American Society of Clinical Oncology/College of American Pathologists guideline recommendations for human epidermal growth factor receptor 2 testing in breast cancer. Arch Pathol Lab Med.

[41] Wolff AC, Hammond MEH, Hicks DG (2013). Recommendations for human epidermal growth factor receptor 2 testing in breast cancer: American Society of Clinical Oncology/College of American Pathologists clinical practice guideline update. Arch Pathol Lab Med.

[42] MD Anderson Cancer Center Residual Cancer Burden Calculator. The University of Texas. www.mdanderson.org/breastcancer_RCB. Published 2018. Accessed 8 June 2018.

[43] Schemper M, Smith TL (1996). A note on quantifying follow-up in studies of failure time. Contemporary Clin Trials.

[44] Pencina MJ, D’Agostino RB (2004). Overall C as a measure of discrimination in survival analysis: model specific population value and confidence interval estimation. Stat Med.

[45] Prat A, Pineda E, Adamo B (2015). Clinical implications of the intrinsic molecular subtypes of breast cancer. Breast.

[46] Sueta A, Yamamoto Y, Hayashi M (2014). Clinical significance of pretherapeutic Ki67 as a predictive parameter for response to neoadjuvant chemotherapy in breast cancer: is it equally useful across tumor subtypes?. Surgery.

[47] Sheri A, Smith I, Johnston S (2014). Residual proliferative cancer burden to predict long-term outcome following neoadjuvant chemotherapy. Ann Oncol.

[48] Liedtke C, Mazouni C, Hess KR (2008). Response to neoadjuvant therapy and long-term survival in patients with triple-negative breast cancer. J Clin Oncol.

[49] Buzdar AU, Ibrahim NK, Francis D (2005). Significantly higher pathologic complete remission rate after neoadjuvant therapy with trastuzumab, paclitaxel, and epirubicin chemotherapy: results of a randomized trial in human epidermal growth factor receptor 2–positive operable breast cancer. J Clin Oncol.

