# Supplementary material for: Validation of Residual Cancer Burden as Prognostic Factor for Breast Cancer Patients After Neoadjuvant Therapy
Source: Ann Surg Oncol. 2019 Aug 26;26(13):4274–83. doi: 10.1245/s10434-019-07741-w (PMC6864028; doi:10.1245/s10434-019-07741-w)

Supporting Figure 1

Recurrence-free survival (RFS) experience of the total study cohort (n=184).

RFS was estimated with a Kaplan-Meier estimator. The 95% confidence band is represented by the grey-shaded area. Numbers below represent a risk table, with the number of RFS events occurring within the respective interval report in round brackets.

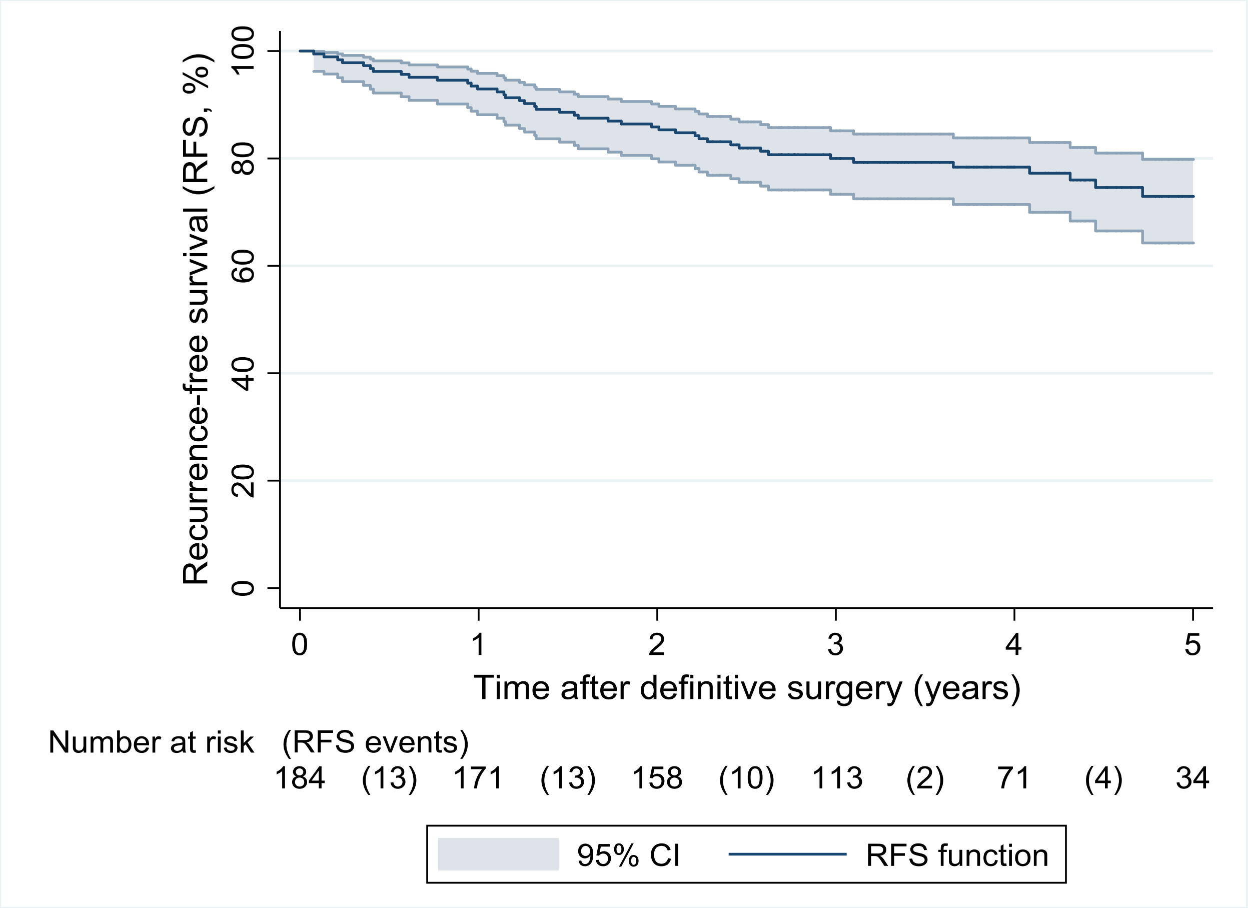

Supplement: Supplementary file 1 — Supplementary material 1 (PDF 120 kb) [file 10434_2019_7741_MOESM1_ESM.pdf]
