# Supplementary material for: Validation of Residual Cancer Burden as Prognostic Factor for Breast Cancer Patients After Neoadjuvant Therapy
Source: Ann Surg Oncol. 2019 Aug 26;26(13):4274–83. doi: 10.1245/s10434-019-07741-w (PMC6864028; doi:10.1245/s10434-019-07741-w)
Supplement: Supplementary file 2 — Supplementary material 2 (PDF 117 kb) [file 10434_2019_7741_MOESM2_ESM.pdf]

Supporting Figure 2

Overall survival (OS) experience of the total study cohort (n=184).

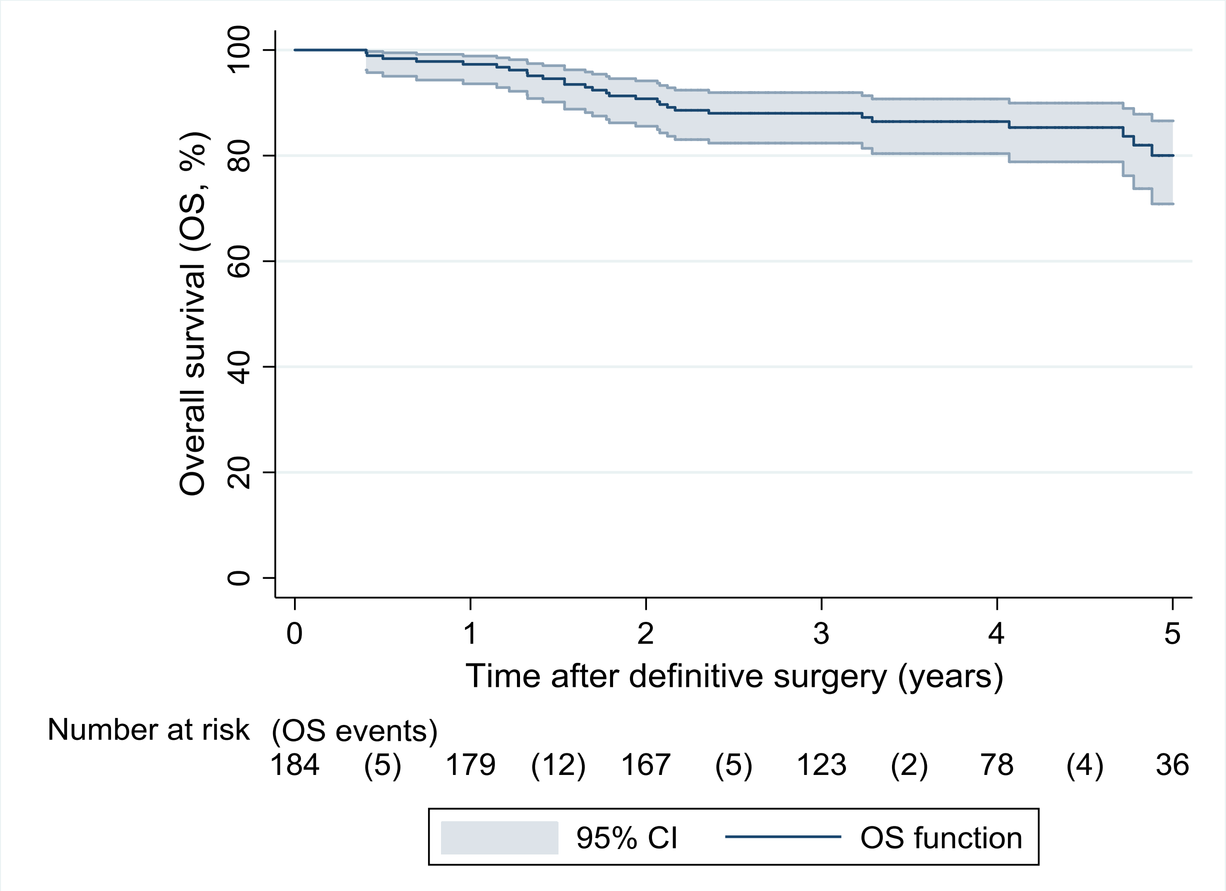

OS was estimated with a Kaplan-Meier estimator. The 95% confidence band is represented by the grey-shaded area. Numbers below the plot represent a risk table, with the number of OS events occurring within the respective interval report in round brackets.
