# Supplementary material for: Validation of Residual Cancer Burden as Prognostic Factor for Breast Cancer Patients After Neoadjuvant Therapy
Source: Ann Surg Oncol. 2019 Aug 26;26(13):4274–83. doi: 10.1245/s10434-019-07741-w (PMC6864028; doi:10.1245/s10434-019-07741-w)
Supplement: Supplementary file 3 — Supplementary material 3 (PDF 171 kb) [file 10434_2019_7741_MOESM3_ESM.pdf]

Supporting Figure 3

Kaplan-Meier Overall survival (OS) functions by RCB class.

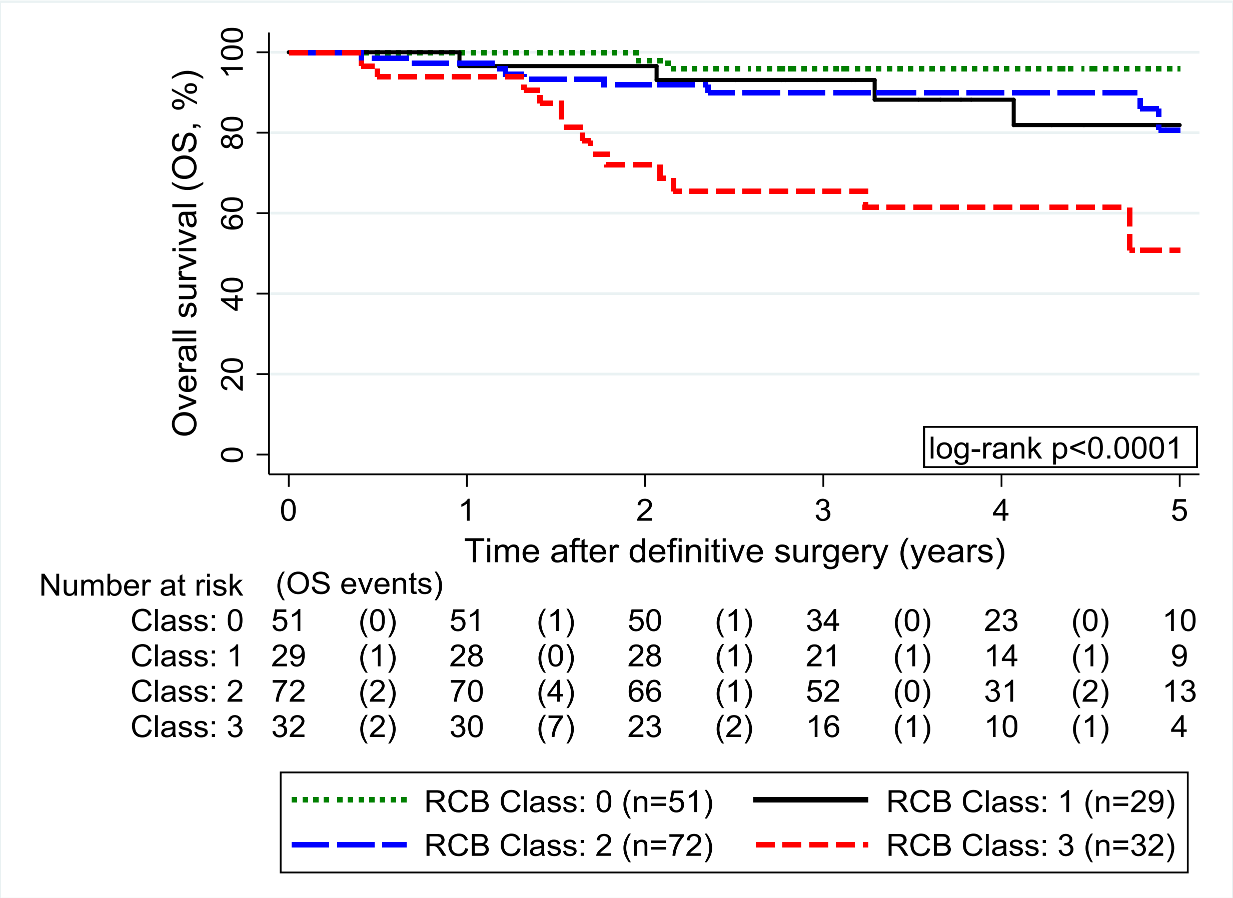

Numbers below the Kaplan-Meier plot represent a risk table per RCB class, with the number of OSS events occurring within the respective interval report in round brackets.
