# Supplementary material for: Validation of Residual Cancer Burden as Prognostic Factor for Breast Cancer Patients After Neoadjuvant Therapy
Source: Ann Surg Oncol. 2019 Aug 26;26(13):4274–83. doi: 10.1245/s10434-019-07741-w (PMC6864028; doi:10.1245/s10434-019-07741-w)
Supplement: Supplementary file 4 — Supplementary material 4 (PDF 34 kb) [file 10434_2019_7741_MOESM4_ESM.pdf]

Supporting Table 1

Outomes by intrinsic subtype

Breast cancer subtype		pCR rate (%, 95%CI)	5-year RFS (%, 95%CI)	5-year OS (%, 95%CI)
ER/PR+ or -/HER2-		7% (2-15)	67% (52-78)	77% (60-87)
HER2+ (classical and variants)		49% (36-62)	79% (65-87)	88% (75-94)
Triple-negative		33% (20-48)	78% (63-88)	79% (61-89)

Abbreviations: 95%CI – 95% confidence interval, ER – estrogen receptor, OS – overall survival, RFS – recurrence-free survival, pCR – pathologic complete remission, PR – progesterone receptor, | - or, HER-2 – human epidermal growth factor receptor 2 (erb-B2).
