# Supplementary material for: Validation of Residual Cancer Burden as Prognostic Factor for Breast Cancer Patients After Neoadjuvant Therapy
Source: Ann Surg Oncol. 2019 Aug 26;26(13):4274–83. doi: 10.1245/s10434-019-07741-w (PMC6864028; doi:10.1245/s10434-019-07741-w)
Supplement: Supplementary file 5 — Supplementary material 5 (PDF 234 kb) [file 10434_2019_7741_MOESM5_ESM.pdf]

Supporting Table 2

A multiple linear regression model investigating predictors of the RCB score (n=184).

The regression coefficient β interpreted as follows: "For one unit increase in the variable, the RCB score changes on average by β." The unit of β is the RCB score.

**Variable**	**Regression coefficient β**	**95%CI**	**p**
Age at entry (per 5 years increase)	0.06	-0.05-0.18	0.278
Molecular breast cancer subtype	/	/	/
---ER+/PR+ or -/ HER2-	Ref.	Ref.	Ref.
---HER2+ (classical and variants)	-1.09	-1.50-(-0.67)	**<0.0001**
---Triple-negative	-0.56	-1.05-(-0.08)	**0.024**
Tumor grade G3	0.11	-0.32-0.54	0.611
Ki-67 labeling index (per 10% increase)	-0.15	-0.23-(-0.07)	**<0.0001**
Postmenopausal status	0.07	-0.44-0.59	0.785
Neoadjuvant systemic therapy – Other regimens than sequential anthracycline + taxane (±anti-Her2)	0.55	-0.10-1.20	0.094

Abbreviations: 95%CI – 95% confidence interval, ER – estrogen receptor, PR – progesterone receptor, | - or, HER-2 – human epidermal growth factor receptor 2 (erb-B2).
