# Supplementary material for: Validation of Residual Cancer Burden as Prognostic Factor for Breast Cancer Patients After Neoadjuvant Therapy
Source: Ann Surg Oncol. 2019 Aug 26;26(13):4274–83. doi: 10.1245/s10434-019-07741-w (PMC6864028; doi:10.1245/s10434-019-07741-w)
Supplement: Supplementary file 6 — Supplementary material 6 (PDF 100 kb) [file 10434_2019_7741_MOESM6_ESM.pdf]

Supporting Table 3

Subgroup analysis: Interactions of RCB with clinically-relevant subgroups.

Note that due to the relatively small number of patients within some selected subgroups, results have to be considered hypothesis-generating. *Other regimens include mostly patients who received only anthracyclines or only taxanes, mainly related to toxicity.

Variable	Subgroups	HR	95%CI	Interaction p-value
**Age**	Age ≤ 65 years	1.54	1.21-1.97	**0.005**
	Age > 65 years	4.50	2.06-9.83	
**Molecular subtype**	ER+PR+ or ER+|PR+ but HER2-	1.75	1.21-2.53	0.333
	HER2+ (classical and variants)	1.82	1.24-2.68	
	Triple-negative	2.46	1.46-4.14	
**Tumor grade**	G1-G2	1.57	0.95-2.58	0.588
	G3	1.86	1.47-2.36	
**Ki-67 labeling index**	> 25%	2.22	1.71-2.89	**0.021**
	≤ 25%	1.24	0.81-1.92	
**Menopausal status**	Pre-/perimenopausal status	1.04	0.68-1.59	**0.004**
	Postmenopausal status	2.20	1.66-2.93	
**Type of systemic treatment**	Other regimens than sequential anthracycline + taxane (±anti-HER2)*	11.98	0.93-153.93	**0.043**
	Sequential anthracycline + taxane (±anti-HER2)*	1.63	1.28-2.06	
**Type of surgery**	Breast conserving therapy	1.53	1.13-2.09	0.211
	Mastectomy	2.05	1.44-2.91	

Abbreviations: RCB – residual cancer burden, HR – hazard ratio, 95%CI – 95% confidence interval, ER – estrogen receptor, PR – progesterone receptor, | - or, HER-2 – human epidermal growth factor receptor 2 (erb-B2).
