# Supplementary material for: Validation of Residual Cancer Burden as Prognostic Factor for Breast Cancer Patients After Neoadjuvant Therapy
Source: Ann Surg Oncol. 2019 Aug 26;26(13):4274–83. doi: 10.1245/s10434-019-07741-w (PMC6864028; doi:10.1245/s10434-019-07741-w)
Supplement: Supplementary file 7 — Supplementary material 7 (PDF 97 kb) [file 10434_2019_7741_MOESM7_ESM.pdf]

Supporting Table 4

Analysis of Overall survival (OS) Univariable Cox models (n=184).

**Variable**	**HR**	**95%CI**	**p**
RCB score (by 1 point increase)	1.96	1.49-2.59	**<0.0001**
RCB class	/	/	/
---0	Ref.	Ref.	Ref.
---1	3.39	0.62-18.50	0.159
---2	3.27	0.71-15.15	0.129
---3	12.89	2.91-57.16	**0.001**
Age at entry (per 5 years increase)	1.13	0.96-1.34	0.135
Molecular breast cancer subtype	/	/	/
---ER+/PR+ or -/ HER2-	Ref.	Ref.	Ref.
---HER2+ (classical and variants)	0.75	0.30-1.91	0.548
---Triple-negative	1.27	0.53-3.01	0.592
Tumor grade G3	2.01	0.81-4.95	0.131
Ki-67 labeling index (per 10% increase)	1.06	0.92-1.21	0.416
Postmenopausal status	1.59	0.73-3.45	0.239
Neoadjuvant systemic therapy – Other regimens than sequential anthracycline + taxane (±anti-Her2)*	3.10	1.25-7.67	**0.014**
Breast conservation	0.64	0.30-1.34	0.234
Axillary lymph node dissection (ALND)	NE	NE	NE
Post-neoadjuvant tumor size (TNM ypT)	/	/	/
---ypTis-ypT0	Ref.	Ref.	Ref.
---ypT1	1.77	0.56-5.56	0.328
---ypT2	5.02	1.51-16.68	**0.002**
---ypT3-ypT4	5.39	1.44-20.11	**0.012**
Number of positive nodes (per 1 increase)	1.13	1.08-1.19	**<0.0001**
Post-neoadjuvant nodal status (TNM ypN)	/	/	/
---ypN0	Ref.	Ref.	Ref.
---ypN1	1.91	0.69-5.30	0.216
---ypN2	3.35	1.10-10.20	**0.033**
---ypN3	7.56	2.71-21.13	**<0.0001**
Adjuvant endocrine therapy	0.57	0.27-1.22	0.148

Abbreviations: HR – hazard ratio, 95%CI – 95% confidence interval, Ref. – Reference category, ER – estrogen receptor, PR – progesterone receptor, | - or, HER-2 – human epidermal growth factor receptor 2 (erb-B2).
